# Synergy effects of copper ion in doxorubicin-based chelate prodrug for cancer chemo-chemodynamic combination therapy

**DOI:** 10.1080/10717544.2023.2219426

**Published:** 2023-06-07

**Authors:** Wen Zhang, Peng Zhang, Xiaopeng Xu, Minghui Li, Shasha Wang, Hongjie Mu, Kaoxiang Sun

**Affiliations:** School of Pharmacy, Key Laboratory of Molecular Pharmacology and Drug Evaluation, (Yantai University), Ministry of Education, Collaborative Innovation Center of Advanced Drug Delivery System and Biotech Drugs in Universities of Shandong, Yantai University, Yantai, P.R. China

**Keywords:** Chemodynamic therapy, chemotherapy, combination therapy, cytotoxicity, antitumor, safety

## Abstract

Doxorubicin (DOX) is a commonly studied chemotherapeutic agent for the treatment of solid tumors, but the severe side effects limit its clinical application. It is shown that DOX-metal chelate has lower *in vitro* cytotoxicity compared with DOX, as the anthracyclines of DOX can form coordinative interaction with transition metal ions. In addition, the transition metal ions could catalyze the production of hydroxyl radicals (·OH) via Fenton/Fenton-like reactions to achieve antitumor chemodynamic therapy (CDT). In this study, copper ions (Cu^2+^) were applied to obtain DOX/Cu(II) prodrug, and a liposomal formulation was used to avoid the rapid blood clearance and optimize the biodistribution of this prodrug. *In vitro* and *in vivo* antitumor results demonstrated that this pH sensitive Cu-chelating prodrug can reduce adverse effects of DOX but improve the antitumor efficiency due to the combination of chemotherapy and chemodynamic therapy. Our study provided a facile and effective approach of metal-chelating prodrug strategy for combination cancer therapy strategy.

## 1. Introduction

Hepatocellular carcinoma (HCC) is the most common primary liver malignant tumor and a leading cause of cancer-related death worldwide (Mo et al., 2020; Mohamed et al., 2021). Despite the enormous achievement in cancer therapy during the past decades (Luerken et al., 2022; Llovet et al., 2021; Yang et al., 2021; Nakajima et al., 2021), chemotherapy is still the main therapeutic modality in the treatment of cancer (Oyama et al., 2021; Wang et al., 2021; Jin et al., 2021). As an anthracycline antibiotic, doxorubicin (DOX) is one of the most important chemotherapeutic agents that frequently used in multiple clinical protocols to treat many kinds of cancer, including HCC (Zheng et al., 2021; Zhou et al., 2022; Pho-Iam et al., 2021). However, the cardiotoxicity and rapid clearance of DOX limit its clinical applications (Cheong et al., 2021; Cagel et al., 2020; Kim et al., 2022; Singh et al., 2022; Phuengkham et al., 2019). Therefore, it’s necessary to develop effective strategies for reducing the toxic side effects of chemotherapeutic agents while enhancing their antitumor efficacy.

A prodrug is a medication which would be converted into a pharmacologically active drug in physiological conditions after it is administered (Lin et al., 2019; Li et al., 2022). It provides a successful tool to reduce the toxic side effects of the anticancer agents, for example albumin-binding prodrug of DOX (Kratz, 2007). In addition, multidrug chemotherapy combination treatment also shows encouraging results in suppressing tumor growth (Zhao et al., 2020; Pereira-Oliveira et al., 2019), but can easily lead to tumor multidrug resistance problem (Tan et al., 2022; Zhang et al., 2022). Thus, it is potential approach to develop a combination strategy to improve therapeutic efficacy and reduce toxicity based on the prodrug principle.

In recent years, chemodynamic therapy (CDT) is becoming an attractive approach for cancer treatment. CDT refers the conversion of endogenous H_2_O_2_ into toxic hydroxyl radicals (·OH) via metal-ion-catalyzed Fenton or Fenton-like reactions at the tumor site, which damages DNA and proteins, thus killing tumor cells and inhibiting tumor growth (Xu et al., 2022; Yu et al., 2021; Li et al., 2023; Su et al., 2022; Yang et al., 2022; Zhou et al., 2022; Xin et al., 2023). Compared with other treatments, CDT has the following advantages: 1) no external energy input requirement; 2) activated by endogenous stimuli (H_2_O_2_ is overexpressed in cancer cells) (Wang et al., 2021; Tang et al., 2019; Zhou et al., 2022; Li et al., 2022). Inspired by the CDT and prodrug principle, we envision that metal-based chemotherapy agent prodrug would be promising tool to achieve synergistic effects in combination with CDT and chemotherapy (Tang et al., 2021; Wang et al., 2019).

**Scheme 1.**
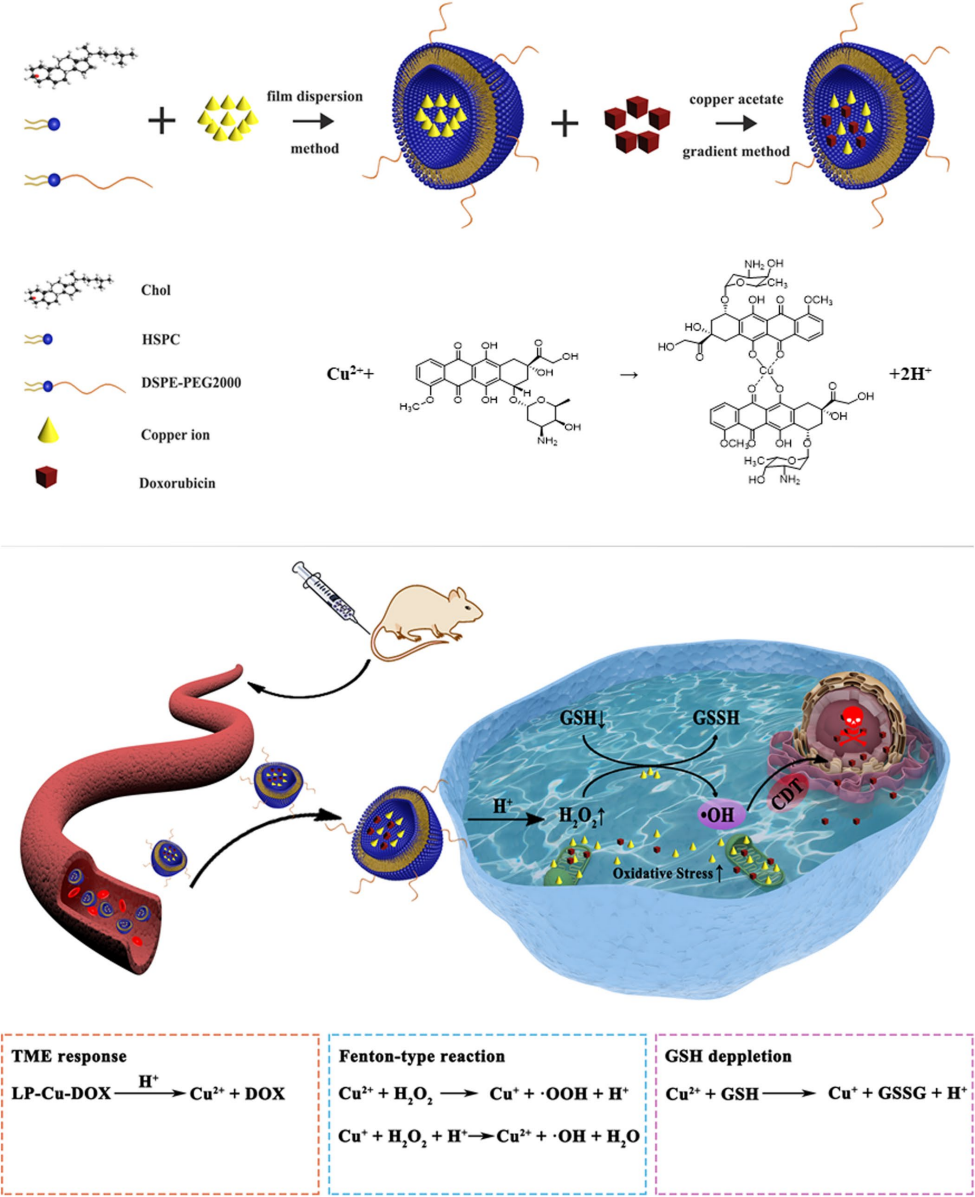
Schematic illustration of the synthesis and antitumor performance of Lp-Cu-DOX

Copper ion is widely used in CDT since Cu^+^ can participate in Fenton-like reaction to generate ·OH (Qiu et al., 2022; Chen et al., 2022; Wang et al., 2022). At the same time, it was also reported that Cu^2+^ can mediate effective loading of DOX, which is based on the complexation of drugs with metal ions. These DOX/Cu(II) chelation with a stability constant of 10^16^ forms in neutral pH could be broke in acidic or biothiol-involved environment, leading to the release of DOX molecules in cancer cells (Yu et al., 2020; Badrooh et al., 2022; Kheirolomoom et al., 2010). However, the high concentration of metal ions may lead to the enhanced toxicity, so the specific dosage remains to be discussed. Additionally, cancer cells are overexpressed glutathione (GSH, 2 ∼ 10 mM), which could defense against oxidative stress by scavenging ROS to maintain the redox balance and strongly reduce the therapeutic effect of CDT (Zhang et al., 2023; Li et al., 2022). Fortunately, Cu^2+^ could deplete the overexpressed GSH and further enhance the therapeutic efficiency of ROS simultaneously (Li & Liu, 2022). Based on the aforementioned information, in this work, a Copper-Dox-chelate prodrug (DOX/Cu(II)) was developed and the *in vitro/vivo* antitumor efficacy and mechanisms of this prodrug were evaluated. To avoid rapid clearance of chemotherapies by the reticuloendothelial system (RES) (Mashreghi et al., 2021), liposomal delivery system was applied in this study, which also could reduce the adverse cardiotoxicity caused by DOX and achieve high enhanced permeability and retention (EPR) -mediated accumulation of therapeutic cargo (shown in Scheme 1). This liposomal formulation, denoted as Lp-Cu-DOX, exhibited good biocompatibility and stability. After taken up by tumor cells, the acidic endosomal environment triggered the dissociation of DOX/Cu(II) to release Cu^2+^ and DOX. Cu^2+^ catalyzed intracellular overexpressed H_2_O_2_ through the Fenton-like reaction to generate abundant ·OH, and consumed the excessive GSH through redox reaction at the same time to further enhance the therapeutic efficiency of CDT. In addition, overloaded Cu^2+^ caused mitochondria injury and thus further enhanced the oxidative stress in tumor cells. This combination of CDT and chemotherapy strategy has shown promise for achieving enhanced cancer treatment outcomes, which has great potential in biomedical application.

## 2. Experimental

### 2.1. Materials and reagents

Hydrogenated soybean phosphotidylcholine (HSPC) was purchased from A.V.T. Pharmaceutical Tech Co., Ltd. (Shanghai, China). Cholesterol (CH), 2′,7′-Dichlorofluorescin diacetate (DCFH-DA), Mitochondrial Membrane Potential Assay Kit with JC-1, 3-(4,5-dimethylthiazol-2-yl)-2,5-diphenyltetrazolium bromide (MTT), Calcein-AM/PI living/dead cell double staining kit, and 4% paraformaldehyde solution were obtained from Beijing Solarbio Science&Technology Co., Ltd. (Beijing, China). 1,2-distearoyl-sn-glycero-3-phosphoethanolamine-N-[methoxy(polyethylene glycol)-2000] (DSPE-PEG2000) was purchased from Shanghai Ponsure Biotechnology Co., Ltd. (Shanghai, China). Doxorubicin hydrochloride (DOX·HCl), copper acetate anhydrous, ammonium acetate, and 3,3′,5,5′-tetramethylbenzidine (TMB) liquid substrate system for ELISA were obtained from Shanghai Macklin Biochemical Co., Ltd. (Shanghai, China). DAPI Fluoromount-G® was purchased from SouthernBiotech (Birmingham, AL, USA). 1,2-bis(2-aminophenoxy)ethane-N,N,N′,N′-tetraacetic acid tetrakis-(acetoxymethyl ester) (BAPTA-AM), GSH (reduced), and 5,5′-dithiobis-(2-nitrobenzoic acid) (DTNB) were purchased from Shanghai Aladdin Biochemical Technology Co., Ltd. (Shanghai, China) Dulbecco’s modified eagle medium (DMEM, high glucose) were obtained from Life Technologies Corporation (Gibco®, USA). Fetal bovine serum (FBS), trypsin solution, penicillin–streptomycin solution, phosphate buffered saline (PBS, pH7.4), Alizarin Red S Staining Kit for Osteogenesis, Annexin V-FITC Apoptosis Detection Kit, and Enhanced ATP Assay Kit were purchased from Beyotime Biotechnology Co., Ltd. (Shanghai, China). MitoTracker® Red CMXRos was obtained from Yeasen Biotechnology Co., Ltd. (Shanghai, China). All other reagents were of analytical grade and used as received.

### 2.2. Preparation of Lp-Cu-DOX

Liposomes encapsulating DOX complex was prepared using the copper acetate concentration gradient method. Briefly, lipids were dissolved in ethanol in a pear-shaped flask, and dried into thin film by rotary evaporation of the organic solvents under vacuum. The lipid molar ratio for all formulations was 2.7:1:0.12 (HSPC: CH: DSPE-PEG2000). The resulting lipid film was hydrated with 4 mL copper acetate solution (80 mg/mL) at 60 °C. The resulting liposomes were homogenized using ultrosonic cell disrupter system at room temperature for 3 min. The unloaded ammonium acetate was removed by dialysis bags (MWCO 14000) at room temperature for 6 h. The copper liposomes were incubated with 1.5 mg/mL DOX solution at 60 °C for 12 h, and the liposomes were dialyzed (MWCO 14000) overnight to remove non-encapsulated DOX at room temperature. Blank liposomes (Lp), DOX-loaded liposomes (Lp-DOX), Cu^2+^-loaded liposomes (Lp-Cu) were prepared by the same method.

### 2.3. Characterization of Lp-Cu-DOX

#### 2.3.1. Encapsulation efficiency (EE) and drug-loading (DL) capacity determination

1 mL methanol was added to a moderate amount of lyophilized Lp-Cu-DOX, then ultrasonically demulsified for 10 min. The copper content of Lp-Cu-DOX was analyzed using inductively coupled plasma mass spectrometry (ICP-MS). The DOX content was determined by high performance liquid chromatography (HPLC), and the encapsulation efficiency and drug loading capacity of DOX was calculated by the following equations:

$$\text{EE}\left(\text{\%}\right)\text{=}\frac{\text{Amount of loaded DOX in liposomes}}{\text{Amount of DOX used in liposomal preparation}}\times \text{100\%}$$

$$\text{DL}\left(\text{\%}\right)\text{=}\frac{\text{Amount}\;\;\text{of}\;\;\text{DOX}\;\;\text{in}\;\;\text{liposomes}}{\text{Total}\;\;\text{amount}\;\;\text{of}\;\;\text{liposomes}}\times \text{100\%}$$

#### 2.3.2. Particle size and zeta potential measurements

The particle sizes of Lp, Lp-Cu, Lp-DOX and Lp-Cu-DOX were respectively measured by dynamic light scattering (DLS) using a particle sizer(BeNano 90 Zeta, Bettersize Instruments Ltd, Dandong City, China). The polydispersity index (PDI) indicates the extent of particle aggregation. The Zeta potential model was used to determine the surface charge of Lp, Lp-Cu, Lp-DOX and Lp-Cu-DOX.

#### 2.3.3. Transmission electron microscopy (TEM) assay

A transmission electron microscope (JEM-1400 Plus; Jeol, Tokyo, Japan) with an accelerating voltage of 120 kV was used for morphologic characterization of the Lp-Cu-DOX. Samples were prepared by dropping 20 μL of the nanocomplex solution on a 200-mesh copper grid with a carbon supporting film and staining with 2% uranyl acetate solution. The grid was dried in air at room temperature before mounting on the sample holder for imaging.

#### 2.3.4. In vitro release investigation of Lp-Cu-DOX in PBS

The *in vitro* release profiles of DOX and Cu^2+^/Cu^+^ from Lp-Cu-DOX were examined using a dialysis method. Briefly, 1 mL of Lp-Cu-DOX was placed in a dialysis bag (MWCO 14000) and immersed in 15 mL of PBS at pH of 7.4, 6.8 and 5.8, which containing Tween-20 (0.5%, v/v). The whole system was in a water bath shaker at 37 °C with continuous shaking at 100 rpm. At predefined time points, 1 mL of PBS was withdrawn and replaced with fresh medium. The concentration of released DOX was determined by HPLC in triplicate. In addition, the concentration of released Cu^2+^/Cu^+^ was determined by Inductively Coupled Plasma Mass Spectrometry (ICP-MS). The release of DOX and Cu^2+^/Cu^+^ was calculated according to the below equation.

$$Er(\%)=\frac{V_{e}\sum_{1}^{n-1}C_{i}+V_{0}C_{n}}{m}\times 100\%$$

where *Er* (%) represents the cumulative release profile of DOX or Cu^2+^/Cu^+^; *V_e_* is the displacement volume of PBS; *V_0_* is the total volume of the in vitro release medium; *C_i_* denotes the drug concentration during the *i*-th displacement sampling; *m* represents the weight of drug loaded.

#### 2.3.5. Detection of in vitro ·OH production

First, 1 mg of lyophilized Lp-Cu-DOX was dispersed in 1 mL of PBS with different pH values (7.4, 6.8, and 5.8), and the mixture was continuously stirred. Next, the supernates were collected by centrifugation and mixed with 1 mL of TMB solution at different time point (0.5, 3, 6, 8, 12h and 24 h). After that, the absorbances of different mixtures were examined by UV-Vis absorbance spectroscopy with a wavelength of 370 nm. And the color changes of the mixture were photographed at various points in time. Then, 1 mg of lyophilized Lp-Cu-DOX was dispersed in 1 mL of PBS with different pH values (7.4, 6.8, and 5.8) and stirred for 24 h. Subsequently, the supernatant was collected by centrifugation and mixed with 1 mL of o-phenylenediamine (OPD) solution. After that, the absorbances of different mixtures were examined by UV-Vis absorbance spectroscopy at the wavelength of 300-600 nm, and the color of the mixture at each pH was also photographed.

#### 2.3.6. In vitro detection of GSH depletion

1 mL Lp-Cu-DOX solutions with different concentrations were dispersed in 1 mL PBS at pH 5.8 and the final concentrations of Lp-Cu-DOX were 20, 40, 60, 80 and 100 μg/mL. The resulting mixture was vigorously agitated on a magnetic agitator for 24 h at 25 °C. Then, various solutions were mixed with 3.5 mL DTNB (4 mM) solution containing 3.5 mL GSH (10 mM). After that, the change of GSH concentration was determined using UV-Vis absorbance spectroscopy at the wavelength of 350-550 nm.

### 2.4. Cell culture and biological evaluation

The human hepatoma (HepG2) cells were used in our cell experiments, and HepG2 cells were cultured in DMEM supplemented with 10% (v/v) heat-inactivated FBS containing 1% penicillin–streptomycin solution at 37 °C with 5% CO_2_ in a humidified incubator. The concentrations of the preparations (unless otherwise specified): (1) Lp-Cu (the Cu^2+^ concentration of 0.125 μg/mL); (2) Lp-DOX (the DOX concentration was 0.5 μg/mL); (3) free DOX (the DOX concentration was 0.5 μg/mL) and (4) Lp-Cu-DOX (the DOX concentration was 0.5 μg/mL, the Cu^2+^ concentration was 0.125 μg/mL), expressed as Lp-Cu-DOX (LC), (5) Lp-Cu-DOX (the DOX concentration was 1.0 μg/mL, the Cu^2+^ concentration was 0.125 μg/mL), expressed as Lp-Cu-DOX (HC).

#### 2.4.1. In vitro cellular uptake evaluation

The cellular uptake of formulations was qualitatively determined by a confocal microscopy (CLSM). HepG2 cells were seeded into confocal dish at 5.0 × 10^4^ (cells/well) with 1 mL of culture medium and cultured overnight. Next, complete medium was replaced with fresh DMEM containing free DOX, Lp-DOX and Lp-Cu-DOX at the DOX concentration of 0.5 μg/mL for various time periods in quadruplicate. After discarding the DOX containing medium, cells were washed three times with PBS and then fixed by 4% paraformaldehyde. Before observation under CLSM, cells were stained with DAPI for 20 ∼ 30 min.

Non-treated cells were used as the control group to quantitatively evaluate the uptake of nanocomplexes by flow cytometry (FCM). After the above treatment, 2 mL of 0.25% trypsin was added to digest the cells in each well and blown with PBS. The cell suspension was centrifuged at 4 °C for 5 min, the supernatant was discarded and 2 mL PBS was added to resuspend the pellet twice, and finally the cells were resuspended in 0.5 mL PBS. All the samples were kept in the ice box and tested by FCM as soon as possible.

#### 2.4.2. In vitro cytotoxicity assay

The *in vitro* cytotoxicity of liposomes was explored by the MTT assays with HepG2 cells. Typically, 200 μL of a HepG2 cell were seeded in a 96-well plate at 5 × 10^3^ cells per well and cultured overnight to fully adhered. Then, the cells were designated to six groups and treated with different samples for 24 h: (1) control (fresh DMEM); (2) Blank Lp (the lyophilized Lp content was 0.14, 1.4, 7, 14, 70, 140, 1400 μg/mL); (3) Lp-Cu (the Cu^2+^ concentration of 0.0025, 0.025, 0.125, 0.25, 1.25, 2.5, 25 μg/mL); (4) free DOX (the DOX concentration of 0.01, 0.1, 0.5, 1, 5, 10, 100 μg/mL); (5) Lp-DOX (the DOX concentration of 0.01, 0.1, 0.5, 1, 5, 10, 100 μg/mL); and (6) Lp-Cu-DOX (the DOX concentration of 0.01, 0.1, 0.5, 1, 5, 10, 100 μg/mL). After carefully aspirated the supernatant, 150 μL of dimethyl sulfoxide (DMSO) was added to each well to dissolve the formed formazan crystals, and the absorbance was measured at 570 nm using a microplate reader (CYT5MV; BioTeK, Winooski, USA) to calculate the relative cell viability. The viability (%) of HepG2 cells was calculated by the below equation. The half-maximal inhibitory concentration (IC_50_) was defined as the DOX concentration required to inhibit cell growth by 50%.

$$\mathrm{Cell}\;\mathrm{viability}(\%)=\frac{{\mathrm{Absorbance}}_{\mathrm{Sample}}}{{\mathrm{Absorbance}}_{\mathrm{Control}}}\times 100\%$$

#### 2.4.3. Apoptosis assay

After incubation, the HepG2 cells were sequentially treated with different samples for 24 h: (1) control (fresh DMEM); (2) Lp-Cu (Cu^2+^ concentration was 0.125 μg/mL); (3) Lp-DOX (C_DOX_ was 0.5 μg/mL); (4) Lp-Cu-DOX (LC); and (5) Lp-Cu-DOX (HC). The cell suspension was centrifuged 5 min at 1500 rpm, then washed twice with PBS. Discarded the supernatant and 500 μL of binding buffer was added to resuspend the cells and 5 μL Annexin V-PITC. 5 μL propidium iodide (PI) was added and the sample was incubated at room temperature in the dark, and the apoptosis rate of cells in each group was observed by FCM.

#### 2.4.4. The live/dead cell viability assay

The cell viability was evaluated using Calcein-AM/PI living/dead double staining kit. HepG2 cells were seeded into confocal dish (5 × 10^4^ cell/well) and cultured overnight. After that, the cells were treated with different samples for 24 h: (1) control (fresh DMEM); (2) Lp (7 μg/mL); (3) Lp-Cu; (4) free DOX; (5) Lp-DOX; and (6) Lp-Cu-DOX. Then cells were collected by centrifugation, and the cell precipitates were resuscitated with 1 × assay buffer. Calcein-AM solution was added to each well and continuously cultured for 25 min, then PI solution (5 μL) was added to cells for 5 min at 37 °C in the dark. Finally, the fluorescence images were recorded by the confocal microscopy. The living cells stained green with Calcein-AM and the dead cells stained red with PI.

#### 2.4.5. Intracellular ROS levels measurement

DCFH-DA assay was used to detect the effect of nanocomplexes on intracellular ROS generation. Cells (5 × 10^4^ cells/well) were seeded in confocal dish overnight and then washed with fresh DMEM. After that, the cells were sequentially treated with different samples for 6, 12, and 24 h: (1) control (fresh DMEM); (2) Lp-Cu; (3) Lp-DOX; (4) Lp-Cu-DOX(LC) with the DOX concentration of 0.5 μg/mL; and (5) Lp-Cu-DOX(HC) the DOX concentration of 1 μg/mL. After the residual nanomaterials were removed, DCFH-DA solution was added and cultured for another 30 min. After the medium was removed, the cells were washed with PBS three times. Finally, the fluorescence images were observed by CLSM.

#### 2.4.6. Mitochondrion membrane potential measurement

Mitochondrial membrane potential detection kit (JC-1) is a rapid and sensitive kit as a fluorescent probe for detecting the change of mitochondrion membrane potential. HepG2 cells were seeded into confocal dish at 5.0 × 10^4^ (cells/well) and cultured overnight and then washed with fresh DMEM. After that, the HepG2 cells were sequentially treated with different samples for 24 h: (1) control (fresh DMEM); (2) Lp-Cu; (3) free DOX; (4) Lp-DOX; and (5) Lp-Cu-DOX. JC-1 solution was added in cells and incubated for 20 min at 37 °C. After removing the medium, the cells were washed with JC-1 staining buffer twice times. Then fixed with 4% paraformaldehyde for 20 min. The cells were washed three times with DMEM and observed by confocal microscopy.

#### 2.4.7. Evaluation of intracellular adenosine triphosphate (ATP) reduction

Enhanced ATP assay kit was used to detect the level of ATP in intracellular. HepG2 cells were seeded into a 6-well plate at 5.0 × 10^4^ (cells/well) and cultured overnight. After that, cells were sequentially treated with Lp-Cu, Lp-DOX and Lp-Cu-DOX for 24 h: (1) control (free DMEM); (2) C1 (the DOX concentration of 0.01 μg/mL, the Cu^2+^ concentration of 0.0025 μg/mL); (3) C2 (the DOX concentration of 0.1 μg/mL, the Cu^2+^ concentration of 0.025 μg/mL); (4) C3 (the DOX concentration of 0.5 μg/mL, the Cu^2+^ concentration of 0.125 μg/mL); (5) C4 (the DOX concentration of 1 μg/mL, the Cu^2+^ concentration of 0.25 μg/mL); (6) C5 (the DOX concentration of 5 μg/mL, the Cu^2+^ concentration of 1.25 μg/mL); and (7) C6 (the DOX concentration of 10 μg/mL, the Cu^2+^ concentration of 2.5 μg/mL). Then all the samples were washed with PBS three times and collected, the ATP level of each group was tested by an Enhanced ATP Assay Kit through a fluorescence spectrophotometer according to the instruction.

### 2.5. In vivo animal studies

BALB/c nude mice (male, 18.0 ∼ 20.0 g, 5 ∼ 6 weeks) were used for the *in vivo* animal studies. All animal experiments and research procedures are in accordance with the corresponding national standards. The HepG2 tumor-bearing nude mice model were established by subcutaneously injection of HepG2 cells (1.0 × 10^7^ cells per mouse) into the right armpit region.

#### 2.5.1. In vivo biodistribution evaluation

The xenograft models of nude mice were intravenously injected with Lp-Cu-DOX (DOX equivalent dosage: 2.5 mg/kg) for 24 h post injection; then, the mice were sacrificed. Major organs (heart, liver, spleen, lung, and kidneys) and tumors were dissected, weighed, and treated with methanol. For quantification of Cu^2+^ in the nude mice, tissue and tumor sections were digested with aqua regia overnight. The biodistributions in organs and tumors were calculated as the DOX and Cu^2+^ percentages of the injected dose per gram of tissues. The concentration of DOX was measured by HPLC, and the concentration of Cu^2+^ was measured by ICP-MS.

#### 2.5.2. In vivo antitumor effect and safety study

When the xenografted HepG2 tumors grew to about 100 mm^3^, the nude mice were randomly divided into five groups (*n* = 6) and treated with different samples via tail intravenously injection every other day: (1) saline; (2) Lp-Cu (Cu^2+^ equivalent dosage: 0.625 mg/kg); (3) free DOX (DOX equivalent dosage: 2.5 mg/kg); (4) Lp-Cu-DOX (LC) (DOX equivalent dosage: 2.5 mg/kg); and (5) Lp-Cu-DOX (HC) (DOX equivalent dosage 5 mg/kg). The therapeutic effect of each group was monitored by measuring the tumor volumes (V) which were calculated by the following equation:

$$V=\frac{\mathrm{Length}\times {\mathrm{Width}}^{2}}{2}$$

Moreover, the tumor sizes and weights of the mice were measured once 2 days to assess the systemic toxicity of virous DOX formulations. The mice were sacrificed on day 21, tumors and major organs were excised, fixed in 4% paraformaldehyde and paraffin embedded. The histological slices were stained with hematoxylin and eosin (H&E), terminal deoxynucleotidyl transferase-mediated dUTP nick-end labeling (TUNEL) and ki67, and then to investigate the antitumor effect and in vivo safety of different formulations.

### 2.6. Statistical Analysis

Quantitative data were indicated as mean ± SD. Means were compared using the student’s t test. Statistical significance was assumed at a value of *p < 0.05, **p < 0.01, ***p < 0.001.

## 3. Results and discussion

### 3.1. Characterization of Lp-Cu-DOX

In this study, UV–vis absorption spectrum was applied to confirm the complexation of DOX and Cu^2+^ (shown in Figure S1). From the spectra that the band at 298 nm (Free DOX) shift to 289 nm (DOX/Cu^2+^) and the band at 528 nm (Free DOX) obviously shift to 499 nm (DOX/Cu^2+^). This blue shift possibly is due to the complexation of DOX and Cu^2+^ disrupts the planarity of chelates (the steric hindrance between tow DOX molecules is increased), and disturbs the charge transfer. Furthermore, the stability of DOX/Cu^2+^ chelation was also confirmed by the UV-vis absorption spectra, no change can be observed from the UV spectra within 1 week, showing a good stability of DOX/Cu^2+^. Cu-DOX codelivery liposomes were successfully prepared by copper acetate concentration gradient method. As shown in Table 1, the entrapment efficiency of the two liposome preparations (Lp-DOX and Lp-Cu-DOX) was 68.67% and 83.83%, respectively. From the results, there are great differences in entrapment efficiency among different preparations. This shows that copper acetate concentration gradient method has a great advantage over ammonium acetate gradient method in liposome encapsulate ability of DOX. This is very likely due to the chelation of DOX with copper ions (DOX/Cu(II), shown in Scheme 1). Since the DOX/Cu(II) is stable (Kheirolomoom et al., 2010) at neutral environment, this chelate could improve DOX loading efficiency and even prevent premature leakage of DOX during systemic circulation. Moreover, this ion-drug chelates exhibit higher dynamic stability over the free ions and drug, which could inhibit the leakage of small molecules or ions diffuse through liquids. It was found that DOX loading capacity was increased with the intraliposomal Cu^2+^ concentration. In all following studies, particles were loaded using 200 mM Cu^2+^ and 3 M DOX, achieving the final ratio of 0.064 mg of DOX and 0.016 mg of Cu^2+^ per milligram of lipid.

**Table 1.** Physicochemical characteristics of liposomes.

	Particle size (nm)	PDI	Zeta potential (mV)	EE (DOX) (%)	DL (DOX) (%)
Lp	209.10 ± 0.29	0.198 ± 0.031	−5.97 ± 0.56	–	–
Lp-Cu	157.93 ± 0.31	0.123 ± 0.012	+15.20 ± 0.70	–	–
Lp-DOX	225.57 ± 1.63	0.181 ± 0.004	−6.03 ± 0.57	68.67 ± 0.45	3.54 ± 0.04
Lp-Cu-DOX	185.66 ± 1.42	0.103 ± 0.009	+13.81 ± 0.31	83.83 ± 1.25	6.39 ± 0.08

**Figure 1.**
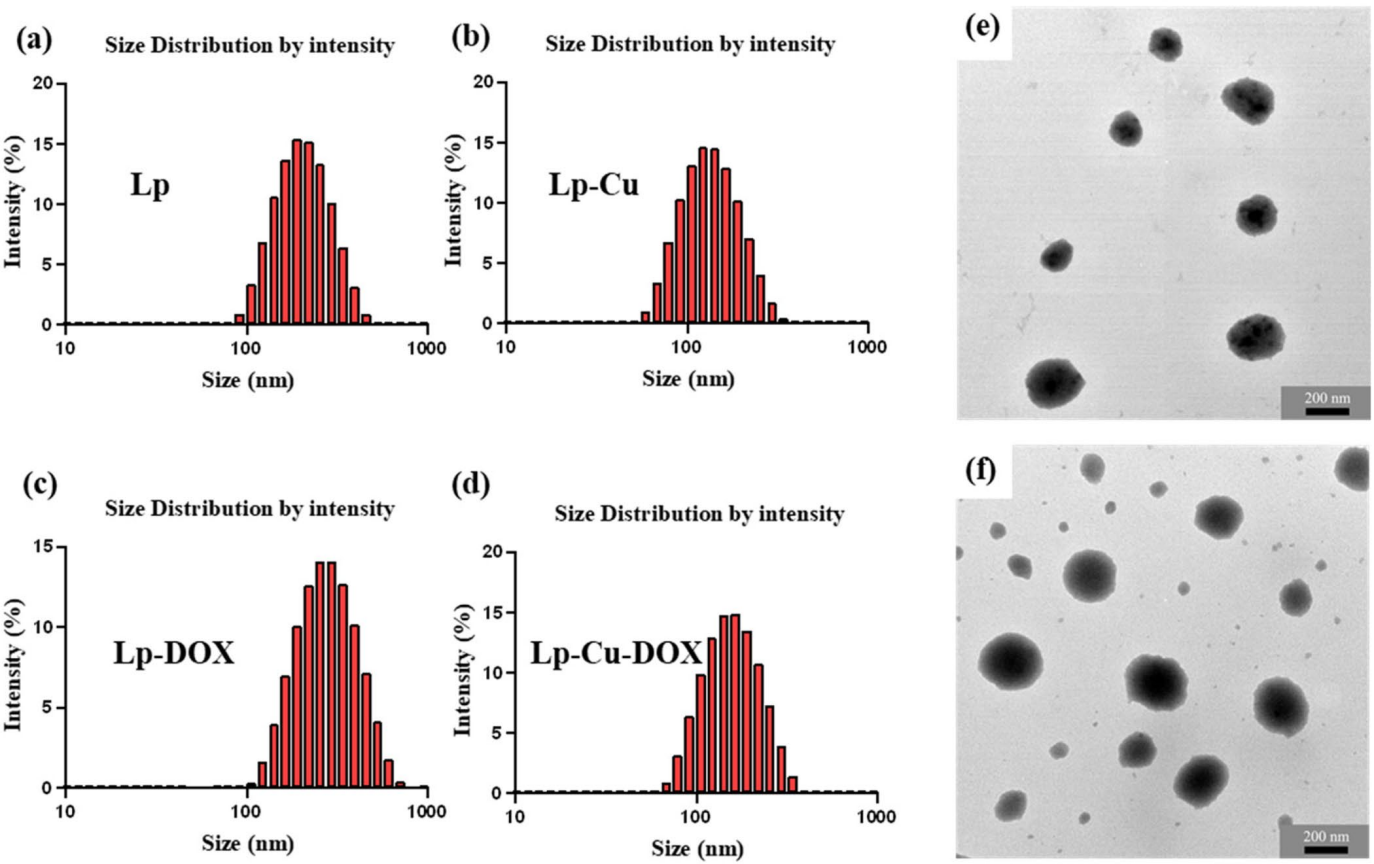
(a–d) Particle sizes distribution of liposomes (a) Lp, (b) Lp-Cu, (c) Lp-DOX and (d) Lp-Cu-DOX, measured at 25 °C. (e–f) TEM images of Lp-Cu-DOX formed after 20 min (e) and 14 days (f).

The particle sizes and zeta potentials of Lp, Lp-Cu, Lp-DOX and Lp-Cu-DOX were shown in Table 1 and particle sizes distribution were shown in Figure 1(a)–(d). The average particle size of Lp was 209.10 ± 0.29 nm, and the average particle size of Lp-DOX was 225.57 ± 1.63 nm. Surprisingly, the introduction of Cu^2+^ could slightly decrease the average particle size of Lps. The average particle sizes of Lp-Cu and Lp-Cu-DOX were 157.93 ± 0.31 nm and 185.66 ± 1.42 nm, respectively. This different particle sizes of liposomes might be mainly attributed to electrostatic forces between phospholipids and Cu^2+^. Due to the electrostatic attraction, negatively charged phospholipids could absorb Cu^2+^ (positive charge) after Cu^2+^ was added into the liposomes system, thereby results in reduced electrostatic repulsive interaction potentials between phospholipids and decreased the particle sizes of Lps. The surface charge of different liposomes could confirm our speculation. The zeta potentials of Lp (-5.97 ± 0.56 mV) and Lp-DOX (-6.03 ± 0.57 mV) were negatively charged, after Cu^2+^ was encapsulated the zeta potential of Lp-Cu (+15.20 ± 0.70 mV) and Lp-Cu-DOX (+13.81 ± 0.31 mV) turned positive charged, which is in line with expectations. Liposomes formed from amphiphiles with negatively charged hydrophobic and hydrophilic moieties are particularly desirable for encapsulating positively charged drugs. Obviously, this provides the possibility of liposomes to increase the drug-loading capacity. Electrostatic repulsion between liposomes is also beneficial for preventing the particles from accumulating, which is important for the storage stability of liposomes.

The morphologies of Lp-Cu-DOX observed by TEM are shown in Figure 1(e,f). The average particle size of Lp-Cu-DOX formed after 20 min was estimated as ∼130 nm from the TEM images. After 14 days, the particle size was increased to ∼230 nm, and no agglomeration of liposomes was observed. The change in particle sizes and morphology results reflected the stability of Lp-Cu-DOX, indicating that the encapsulation of Cu^2+^ could help improve the stability of liposomes.

#### 3.1.1. In vitro release investigation

**Figure 2.**
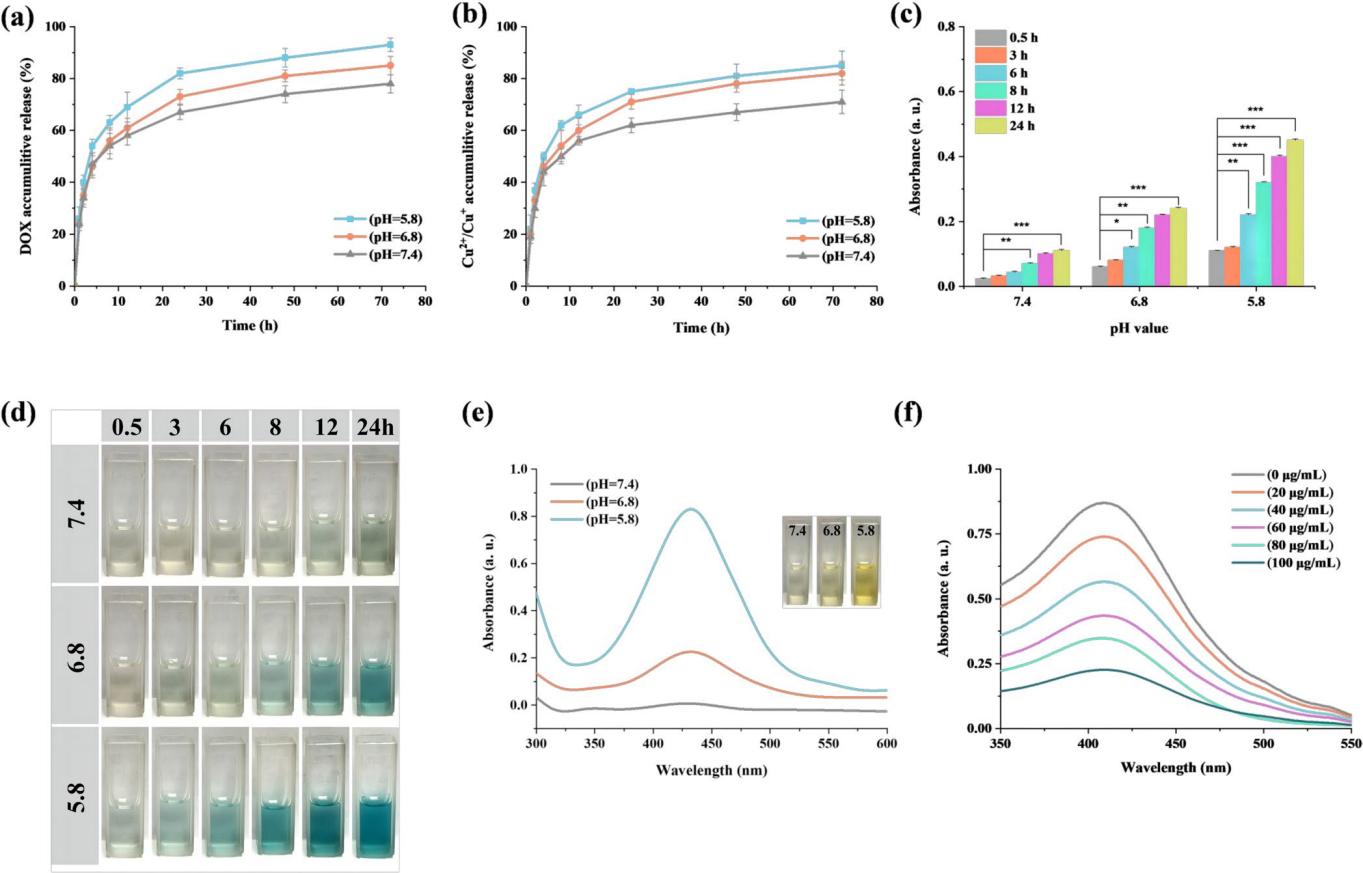
Lp-Cu-DOX in vitro release study: (a) cumulative release of DOX from Lp-Cu-DOX and (b) cumulative release of Cu^2+^/Cu^+^ from Lp-Cu-DOX. (c)Time-dependent absorbance at 370 nm wavelength and (d) color change of TMB solution under different conditions. (e) UV–Vis absorption spectra of OPD solution mixed with various Lp-Cu-DOX solutions after different treatments (insets: digital pictures of different samples). (f) GSH depleting ability of Lp-Cu-DOX.

In vitro release profiles of DOX and Cu^2+^/Cu^+^ from Lp-Cu-DOX in PBS buffer at 37 °C is shown in Figure 2(a,b). It could be seen that the release tendency of DOX and Cu^2+^/Cu^+^ from Lp-Cu-DOX were similar, suggesting that the time and space challenge of dual-drug delivery might be solved by liposomal formulation. It was found that about 81% of DOX had been released from Lp-Cu-DOX at pH 5.8 at 24 h, whereas 73% and 67% of DOX was released at pH 6.8 and 7.4, respectively. And the cumulative release of Cu^2+^/Cu^+^ was 75%, 71%, and 62% at pH 5.8, 6.8, and 7.4, respectively, within 24 h. Furthermore, the percentage of Cu^2+^/Cu^+^ cumulative release was 85%, 82%, and 71% at pH 5.8, 6.8, and 7.4, respectively, within 72 h (Figure. 2b). The faster DOX and Cu^2+^/Cu^+^ release at pH 5.8 may be due to that the DOX/Cu(II) chelate was sensitive to H^+^, and the chelate dissociated rapidly in H^+^-rich environment to achieve the fast release the DOX and Cu^2+^.

#### 3.1.2. Detection of in vitro ·OH production

Since the mechanism of CDT is through generation of ROS by Fenton-like chemistry, it’s necessary to verify the hydroxyl radicals generate ability by Lp-Cu-DOX. In this study, the generation of ·OH was monitored using TMB substrate. The TMB could be oxidized to oxidized TMB (oxTMB) by ·OH, which had a strong absorbance at approximately 370 nm, and this process caused rapid color change from colorless to blue. As shown in Figure 2(c), the absorbance intensity of TMB solution increased with the extension of interact time and it was accompanied by a visible color change, indicating the formation of ·OH. Not surprisingly, the Lp-Cu-DOX led to the most obvious color change of TMB in a mildly acidic environment (pH 5.8) (Figure 2d). ·OH production ability under different conditions of pH were tested, and Lp-Cu-DOX in pH 5.8 had the strongest absorption at 450 nm (characteristic wavelength of OPD) (Figure 2e). This is because acidic environment (H^+^ rich) can promote Cu^+^-initiated Fenton-like reaction and production of ·OH (Cu^+^ + H_2_O_2_ + H^+^ → Cu^2+^ + ·OH + H_2_O). Considering the acidic intracellular microenvironment of tumor cells and H^+^ production during GSH depletion process (which is discussed later), the production of ·OH and tumor cells killing effect can greatly be improved in biological evaluation and practical use.

#### 3.1.3. In vitro GSH depletion

The overexpressed GSH causes the consumption of ROS, leading to diminished CDT antitumor effect. In Lp-Cu-DOX system, copper ions not only promoted the generation of ·OH, but also accelerated GSH consumption sequentially resulted in enhanced CDT. The *in vitro* GSH depletion ability of Lp-Cu-DOX was verified by Ellman’s assay. Based on the free radical-scavenging effect and strong combination with Cu^2+^ of GSH, a detection method for GSH using the Lp-Cu-DOX-DTNB was established. The correlation between alterations in absorbance in 412 nm (characteristic wavelength of GSH) with the change in Lp-Cu-DOX concentration were tracked to evaluate the GSH consumption ability. Although acidic conditions are not favorable for the GSH depletion from the chemical reaction (Cu^2+^ + GSH → Cu^+^ + GSSH + H^+^), H^+^-promoted Fenton-like reaction could accelerate oxidation of Cu^+^ to Cu^2+^, further facilitating the GSH depletion. Furthermore, GSH depletion measurement at the 24 h timepoint revealed the concentration-dependence of Lp-Cu-DOX and GSH depletion ability. As displayed in Figure 2(f), with the increasing concentration of Lp-Cu-DOX, the absorbance at 412 nm gradually diminished after 24 h because the interaction between Cu^2+^ and GSH. The robust GSH depletion ability of Lp-Cu-DOX is beneficial for lessening the quenching of ROS, which amplify the CDT effect.

### 3.2. In vitro biological evaluation of Lp-Cu-DOX

#### 3.2.1. In vitro cellular uptake evaluation

**Figure 3.**
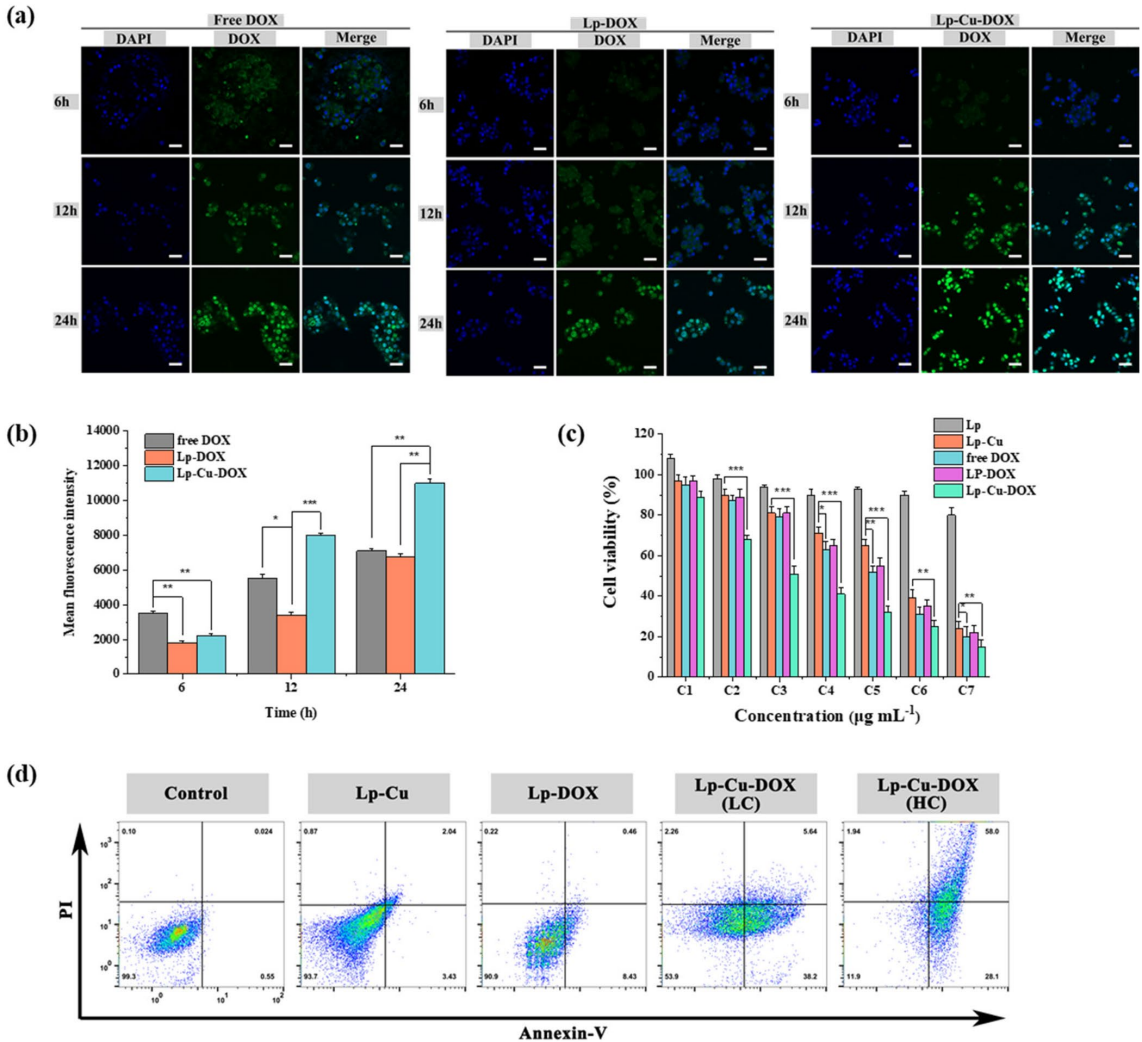
(a) Qualitative cellular uptake and (b) quantitative cellular uptake of HepG2 cells incubated with free DOX, Lp-DOX and Lp-Cu-DOX at 37 °C for different times (6, 12 and 24 h). Scale bar: 25 μm. (c) Cell viabilities of HepG2 cells incubated with different concentrations of Lp, Lp-Cu, free DOX, Lp-DOX and Lp-Cu-DOX. (d) cell apoptosis ratios determined by FCM of HepG2 cells incubated with different treatments.

The cellular uptake behaviors of free DOX, Lp-DOX and Lp-Cu-DOX were investigated by confocal microscopy to investigate the effect of the phospholipid shell layer and surface charge on the internalization capability of the nanocomposites. At the first 6 h, green fluorescence intensity emitted by DOX from free DOX group was stronger than the other formulations (Figure 3a). This can be explained from the distinct uptake pathways of DOX that free DOX entered into cells rapidly via the passive diffusion mechanism. Moreover, compared with obvious green fluorescence signal in Lp-Cu-DOX, nearly no DOX green fluorescence was detected in Lp-DOX group (at 6 h), which was due to the enhanced affinity by electrostatic attractions between positively charged Lp-Cu-DOX and negative HepG2 cell membranes. As expected, the green fluorescence enhanced with increasing treatment time, which indicated the time-dependent uptake behavior of Lp-Cu-DOX by HepG2 cells.

To further verify this result, the cellular uptake of DOX from different formulations was quantitatively analyzed by FCM. As shown in Figure 3(b), the mean fluorescence intensity increased with the extension of incubation time up to 24 h, which confirmed that the uptake of Lp-Cu-DOX by HepG2 cells was time-dependent. Cellular uptake evaluation results indicate that the positive surface charge played key roles in the cellular internalization process with facilitating the uptake of liposomes in the in vitro environment.

#### 3.2.2. In vitro cytotoxicity assay

MTT assay was utilized to evaluate the in vitro toxicity of various formulations on HepG2 cells. The IC_50_ of Lp-Cu, free DOX, Lp-DOX and Lp-Cu-DOX against HepG2 cells at 24 h were 2.03 ± 3.94, 3.973 ± 1.03, 5.155 ± 3.15 and 0.64 ± 2.57 μg/mL (Figure S4). Apparently, HepG2 cells were more sensitive to DOX than Cu^2+^ when they were applied alone. After the combined application, however, the antitumor effect was greatly improved. Moreover, a blank nanocarrier without DOX or Cu^2+^ (Lp) showed minimal cytotoxicity even at the high concentration (100 μg/mL) tested, which indicated excellent biocompatibility of the phospholipid material. Furthermore, cell viability decreased significantly from 89.96% to 14.86% when the DOX equivalent concentration increased from 0.01 to 100 μg/mL for Lp-Cu-DOX (Figure 3c). These cytotoxicity results demonstrated that the Lp-Cu-DOX, in contrast to the free DOX group, could dramatically promote toxicity at all tested concentrations against HepG2 cells, which implied that the enhanced cytotoxicity was not only attributed to the improved cellular uptake of DOX, but also because the hydroxyl radicals from the Fenton-like reaction mediated by Cu^2+^. The cytotoxicity results suggest that CDT strategy combined with chemotherapy drugs, such as DOX, demonstrate a synergistic antitumor effect.

#### 3.2.3. Apoptosis analysis and live/dead cell staining assay

To determine whether Lp-Cu-DOX induced apoptosis, the apoptosis ratios of HepG2 cells with different treatments were examined by FCM (results were shown in Figure 3d). From the results, apoptotic rates in combination group were markedly increased than that in the single treatment group. HepG2 cells in each group could be induced to apoptosis after intervening 24 h, which was significantly different from the blank serum group. However, only few apoptotic HepG2 cells were detected in treated group with Lp-Cu (5.47%) and Lp-DOX (8.89%). Lp-Cu-DOX treated group resulted in a higher proportion of apoptotic cells when compared to that in the single treated group. Under the same DOX concentration, the apoptosis ratio of Lp-Cu-DOX (LC) treated HepG2 cells 43.8%, which was obviously higher than that of Lp-DOX treated group. Similar results were obtained in the Lp-Cu group. Moreover, Lp-Cu-DOX induced apoptosis of HepG2 cells in a DOX-concentration dependent manner. Both Lp-Cu-DOX (HC) and Lp-Cu-DOX (LC) had the same concentration of Cu^2+^, however, the concentration of DOX from Lp-Cu-DOX (HC) was 1 μg/mL, it induced a much higher apoptosis ratio (86.1%) compared with Lp-Cu-DOX (LC), which final DOX concentration was 0.5 μg/mL.

**Figure 4.**
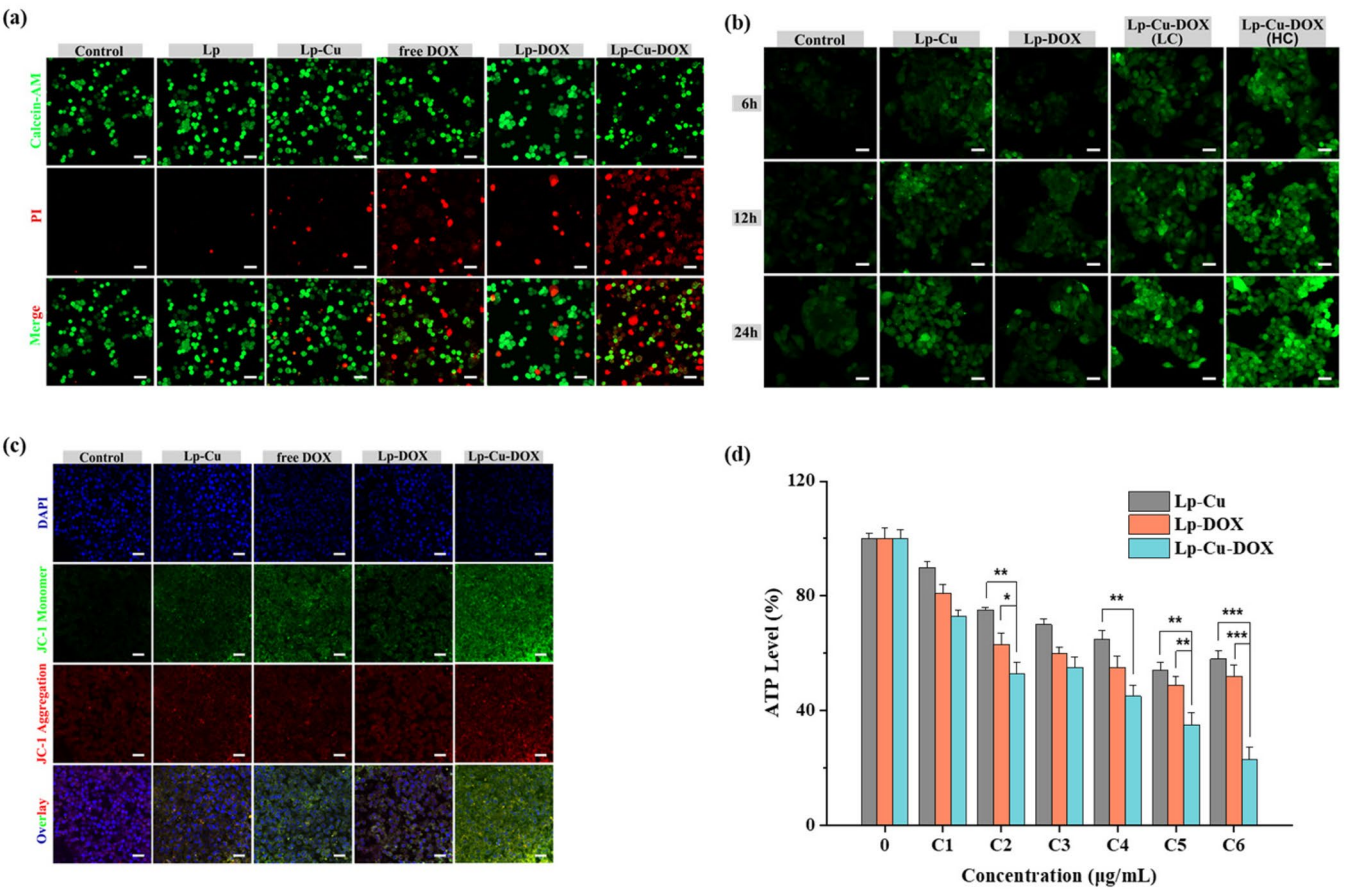
(a) Calcein-AM/PI staining of HepG2 cultured 24 h with different treatments. (b) Fluorescent images of DCFH-DA assays of HepG2 cells incubated with different treatments. (c) Fluorescent images of JC-1assays of HepG2 cells under different conditions. Scale bar: 25 μm. (d) ATP level of HepG2 cells after incubation with different treatments (*n* = 3).

To qualitatively verify the results of the above test, cells were also observed after live/dead staining, which live cells were marked with green and dead cells with red (Figure 4a). Similar to MTT results, it was observed that green-stained living cells were dominant in Lp and Lp-Cu group after 24 h compared with the control group. This result not only proved that the carrier material had great biocompatibility, but also showed that Cu^2+^ alone could not produce obvious CDT antitumor effect, even Cu^2+^ induced generation of ·OH by Fenton-like catalytic reaction. While red-stained dead cells were obvious in DOX group, but decreased significantly after encapsulated with Lp, indicating that the liposome inhibited the rapid release of DOX to decrease the antitumor ability within a short period of time. Red-stained dead cells were dominant in Lp-Cu-DOX group, which confirmed that the combined application of Cu^2+^ and DOX can greatly enhance the antitumor effect. Both the apoptosis analysis and Live/Dead cell staining results confirmed that the DOX/Cu(II) chelate didn’t affect the antitumor of any single component, and the cancer cell toxicity of Lp-Cu-DOX was related to the synergistic effect of chemotherapy (DOX) and chemodynamic therapy (Cu^2+^).

#### 3.2.4. Measurement of intracellular ·OH level

Though the *in vitro* generation of ·OH were investigated by TMB method, it’s still important to confirm the ·OH condition at the cell level. DCFH-DA was employed as a detector to verify the intracellular ·OH level. As can be seen in Figure 4(b), no obvious green fluorescence signal of 2′,7′-dichlorofluorescein (DCF) was presented in HepG2 cells incubated with Lp-DOX, while limited green fluorescence was observed after HepG2 cells were treated with Lp-Cu. That was because Cu^2+^ could catalyze the overexpressed H_2_O_2_ in tumor cells to produce ·OH, resulting in cell oxidative stress. HepG2 cells incubated with Lp-Cu-DOX exhibited significantly stronger ·OH signal than those observed in other conditions, and the fluorescence intensity was further enhanced with the prolongation of the administration time. It is worth noting that the green fluorescence of HepG2 cells incubated with Lp-Cu-DOX (low DOX concentration) presented a measure of quenching compared with Lp-Cu-DOX (high DOX concentration). Though it has been suggested that DOX could induct ROS production to induce apoptosis of tumor cells, Lp-Cu and Lp-Cu-DOX treated groups exhibited stronger ·OH signal than Lp-DOX, confirming the synergistic enhanced ·OH generation ability of Cu^2+^ and DOX. A positive relationship existed between the increased intracellular ·OH level and high levels of apoptosis induced by Lp-Cu-DOX, suggesting that the Cu-DOX exerts synergistically enhanced cytotoxic effect in tumor cells with elevated ·OH levels combined with DOX.

#### 3.2.5. Mitochondrion membrane potential measurement

Mitochondrial damage and cell early apoptosis are usually associated with a decrease of mitochondrial membrane potential, which was monitored using JC-1 dye. High membrane potential (healthy cells) makes JC-1 to form red aggregates, but in low membrane potential mitochondria (apoptotic cells), JC-1 forms green monomers. As shown in Figure 4(c), Lp-Cu and Lp-DOX treated groups exhibited similar green fluorescence signal, while free DOX showed rather strong green fluorescence level. In contrast, HepG2 cells treated with Lp-Cu-DOX exhibited the most significant green fluorescence signal (the declined mitochondrial membrane potential), suggesting the cells were in the early phase of apoptosis. It manifested that Lp-Cu-DOX could trigger the accumulation of intracellular ·OH in HepG2 cells through Cu^2+^-mediated enhancement of oxidative stress combined with DOX, which in turn presented severe mitochondrial damage of HepG2 cells.

#### 3.2.6. Evaluation of intracellular ATP reduction

In order to further verify the damage of HepG2 cell mitochondria, enhanced ATP assay kit was used to detect the level of intracellular ATP. Since mitochondria are the major site of ATP production, the level of intracellular ATP level could reflect the status of mitochondrial function. As shown in Figure 4(d), the ATP content of Lp-Cu and Lp-DOX at the concentration of C6 was slightly decreased by 52.3% and 58.6% compared with the control group. However, the intracellular ATP content of HepG2 cells was significantly decreased about 76.9% after treated with Lp-Cu-DOX under the concentration of C6. At the same time, ATP level significantly decreased with increasing DOX concentration compared with Lp-Cu, suggesting that the introduction of DOX synergistically improved the oxidative stress with Cu^2+^-mediated CDT, thus caused the dysfunction of the mitochondria and further lead to HepG2 cells apoptosis.

## 4. *In vivo* animal studies

### 4.1. In vivo biodistribution evaluation

**Figure 5.**
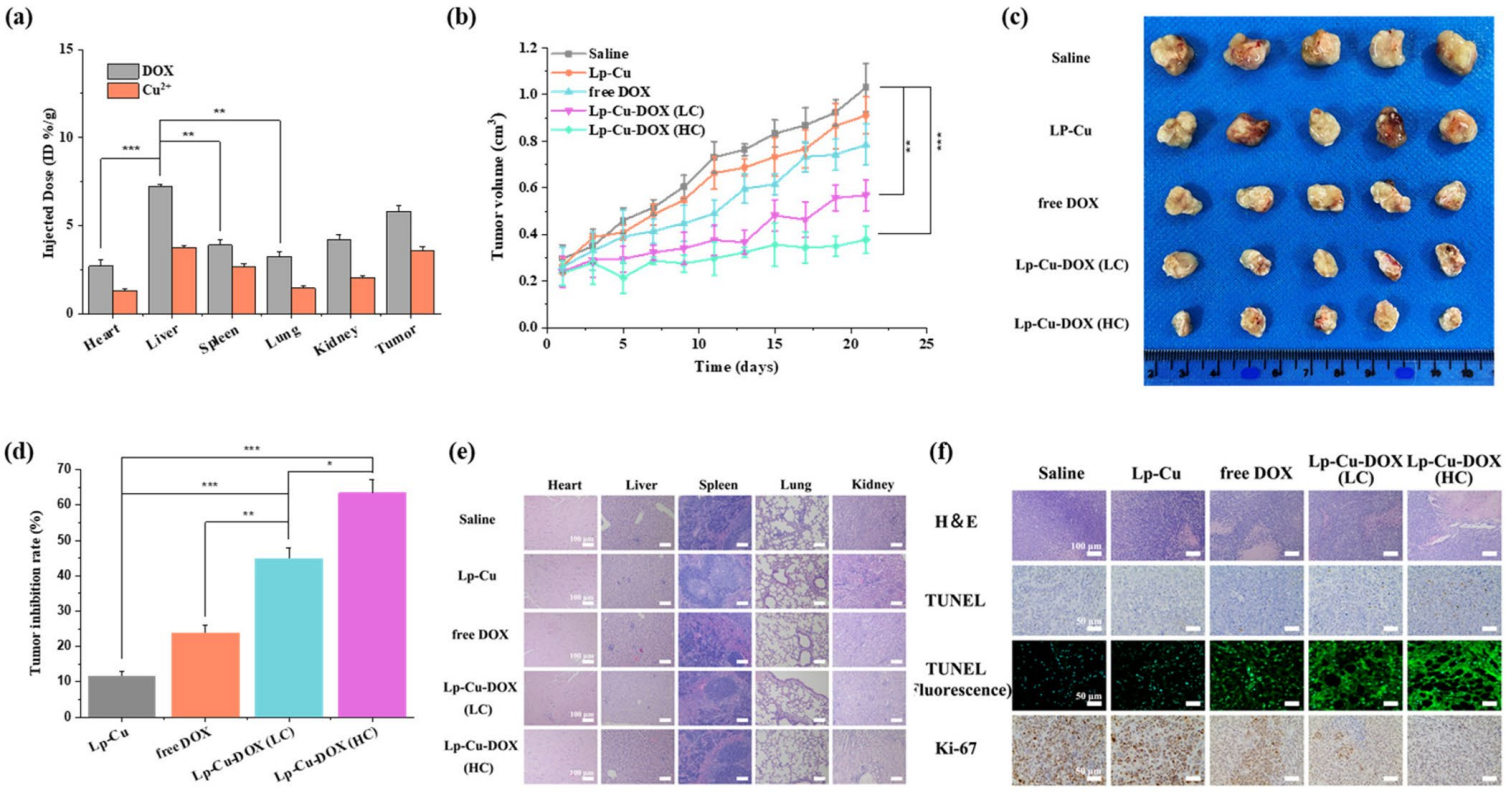
(a) the biodistribution of DOX and Cu^2+^ (injected dose (ID) % of DOX and Cu^2+^ per gram of tissues) in main tissues and tumor in 24 h of intravenous Administrations of Lp-Cu-DOX (*n* = 5). (b) tumor volume (cm^3^) of different formulations. (c) Photograph of tumors dissected from each group at 21st day. (d) Tumor inhibition rate (%) of different formulations. (e) H&E staining of major organs (heart, liver, spleen, lung and kidney) from the mice body after treatment with different groups after 21 days treatment. Scale bar: 100 μm. (f) H&E, TUNEL, TUNEL (fluorescence) and Ki-67 staining images of tumor tissue sections obtained from tumor-bearing mice with various treatments.

The *in vivo* biodistribution behavior of Lp-Cu-DOX in the main organs and tumor tissue of tumor-bearing nude mice was determined by measuring the concentration of DOX and copper ions in each tissue. From results shown in Figure 5(a), after intravenous injection for 24 h, the detected tissue level of DOX and Cu^2+^ from Lp-Cu-DOX had the same biodistribution trend, suggesting that the DOX/Cu(II) chelate prodrug and liposomal carrier system could solve the dual-delivery inconsistent pharmacokinetics limitation of small molecules and ions in vivo. It was found that the accumulation of DOX in the heart tissue was 2.6% ID/g (injected dose/per gram organ), indicating that Lp-Cu-DOX had the therapeutic potential for reducing the cardiotoxicity of DOX. In addition, the concentration of DOX in the liver for 24 h was 4.7%, which likely due to the rapidly taken up by macrophages thus causing the accumulation of liposomes in the liver. The tumor accumulations of DOX and Cu^2+^ maintained at a high level of 5.8% and 3.6% ID/g, respectively. There are two main reasons for this high accumulation, firstly, liposomes could accumulate within tumor tissue via EPR-mediated passive diffusion; secondly, the enhanced affinity between positively charged Lp-Cu-DOX and negative HepG2 cell membranes, concentrating the drug in tumor sites.

### 4.2. In vivo antitumor effect and safety study

The in vivo antitumor effect of Lp-Cu-DOX was evaluated in details to further verify the synergistic therapeutic efficacy of chemotherapy combined with CDT strategy. As shown in Figure 5(b), tumor growth inhibition was observed in the Lp-Cu and free DOX treated group compare with saline group, which indicated the limited antitumor therapy based on the Cu^+^-mediated Fenton-like reaction. Excitingly, both Lp-Cu-DOX (LC) and Lp-Cu-DOX (HC) treatment groups showed significant inhibition on tumor growth, with tumor volume substantially smaller than the control group. The digital photographs of tumor-bearing mice and tumor tissues in various groups also revealed the tumor inhibition ability of Lp-Cu-DOX (Figures S5 and 5c). After 21 days treatment, the tumors of nude mice were harvested and weighted for evaluating the tumor growth inhibition rate. Compared with tumor suppression rates in DOX of 23.77% or suppression rates in Lp-Cu of 11.58%, tumor suppression rates of the Lp-Cu-DOX (LC) and Lp-Cu-DOX (HC) reached 44.89% and 63.36% (Figure 5d). During in vivo animal studies, there was no significant difference in body weight change among the groups (Figure S6). To further evaluate the in vivo safety of Lp-Cu-DOX, the histological analysis of the main organs was performed and shown in Figure 5(e). There were no apparent damages in the detected organs from Lp-Cu-DOX, confirming its good biosafety. However, from the stained major organs slices images, a certain degree of tissue damage or inflammatory cell infiltration was detected in the free DOX treatment. As a contrast, insignificant inflammation and negligible apoptotic/necrotic cells were found in Lp-Cu-DOX treated group.

To further determine the mechanism for tumor inhibition, tumor sections were analyzed by H&E staining and immunohistology (Figure 5f). From the H&E staining results, it was found that most tumor nuclei showed highly pleomorphic hyperchromatic and high mitotic activity in Lp-Cu, free DOX or Lp-Cu-DOX (LC) treated group. In contrast, intensive necrosis area stained by eosin was dominated in Lp-Cu-DOX (HC) treated group and pyknosis can also be observed, resulting from irreversible condensation of chromation in the nuclei of tumor cells undergoing necrosis or apoptosis. This observation suggests the synergistically enhanced antitumor effect of Cu^2+^ and DOX combination.

In addition, TUNEL staining images revealed negligible level of necrotic cells was observed in saline-treated or Lp-Cu-treated group. The apoptotic cells were remarkably increased in the tumor tissues from the free DOX group and Lp-Cu compared with the control group. In particularly, tumors cells presented almost completely necrotic status in Lp-Cu-DOX (HC) group, indicating the promising antitumor efficacy. The similar result could also be seen in TUNEL(fluorescence) staining image. In the TUNEL(fluorescence) assay, the nuclei of TUNEL-positive (apoptotic) cells appeared green, indicating apoptotic cells. A significantly bright green fluorescence signal was observed in Lp-Cu-DOX group compared with the other groups, confirming an abundance of apoptotic cells in the Lp-Cu-DOX treated tumor-bearing nude mice. Meanwhile, the degree of tumor proliferation was determined by Ki67 staining. Ki67 is an indicator of tumor replication, high level of Ki67 usually indicate a rapid tumor growth rate. From the result, cells in control group and Lp-Cu group exhibited high Ki67 staining, while Lp-Cu-DOX groups had fewer Ki67 positive cells, indicating effectively inhibited tumor growth. All these findings are consistent with our observations above, which suggesting the strong tumor inhibitory ability of Lp-Cu-DOX was due to the chemotherapy – chemodynamic therapy combination strategy.

## 5. Conclusions

In summary, we have designed and prepared a chemo-chemodynamic combination therapeutic nanomedicine, Lp-Cu-DOX. Lp-Cu-DOX could effectively catalyze H_2_O_2_ into ·OH via Cu-triggered Fenton-like reaction. Meanwhile, the acidic condition could accelerate the release of DOX and Cu^2+^ compared with neutral pH, demonstrating that Lp-Cu-DOX was a pH-promoted combination therapeutic system. In addition, both *in vitro* and *in vivo* studies confirm that Lp-Cu-DOX exhibit an excellent anticancer efficacy against HepG2 cells. The in vivo anticancer study showed that Lp-Cu-DOX had an ideal biodistribution behavior and tumor inhibition ability with minimal side effects. Our study thus provides a chemo-chemodynamic combination therapeutic strategy for cancer therapy. Since chemotherapeutic agent can be replaced by photosensitizers or sonosensitizers, more different types of combination antitumor strategies such as chemo-photodynamic or chemo-sonodynamic therapy can be achieved.

## Supplementary Material

Supplemental Material
