# Supplementary material for: Synergy effects of copper ion in doxorubicin-based chelate prodrug for cancer chemo-chemodynamic combination therapy
Source: Drug Deliv. 2023 Jun 7;30(1):2219426. doi: 10.1080/10717544.2023.2219426 (PMC10249455; doi:10.1080/10717544.2023.2219426)
Supplement: Supplemental Material [file IDRD_A_2219426_SM5423.docx]

Supporting Information for

**Synergy effects of copper ion in doxorubicin-based chelate prodrug for cancer chemo-chemodynamic combination therapy**





**Figure S1.** The UV-visible absorption spectra of free DOX, Cu^2+^, DOX/ Cu^2+^ (1h) and DOX/ Cu^2+^ (7 d).





**Figure S2.** The UV-visible absorption spectra of free DOX, Cu^2+^, DOX/ Cu^2+^ and DOX/ Cu^2+^ (pH=5).


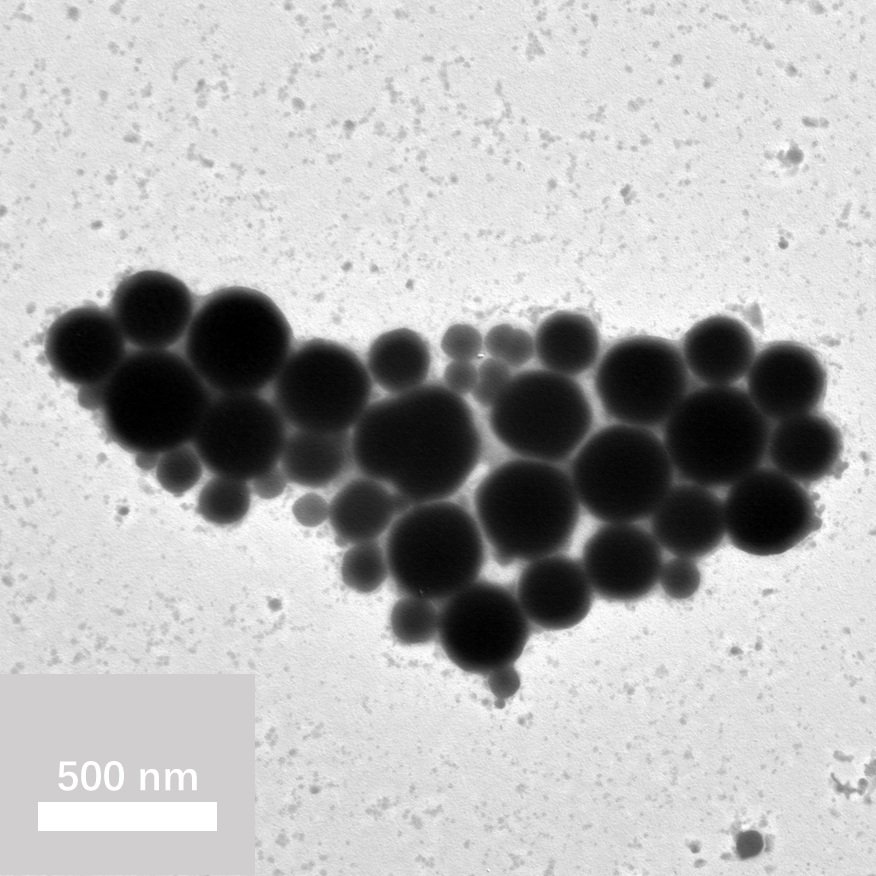


**Figure S3.** TEM images of fresh Lp-Cu-DOX (unlyophilized).

**Figure S4** IC50 (μg/mL) of different concentrations of Lp, Lp-Cu, free DOX, Lp-DOX and Lp-Cu-DOX on HepG2 cells for 24 h.


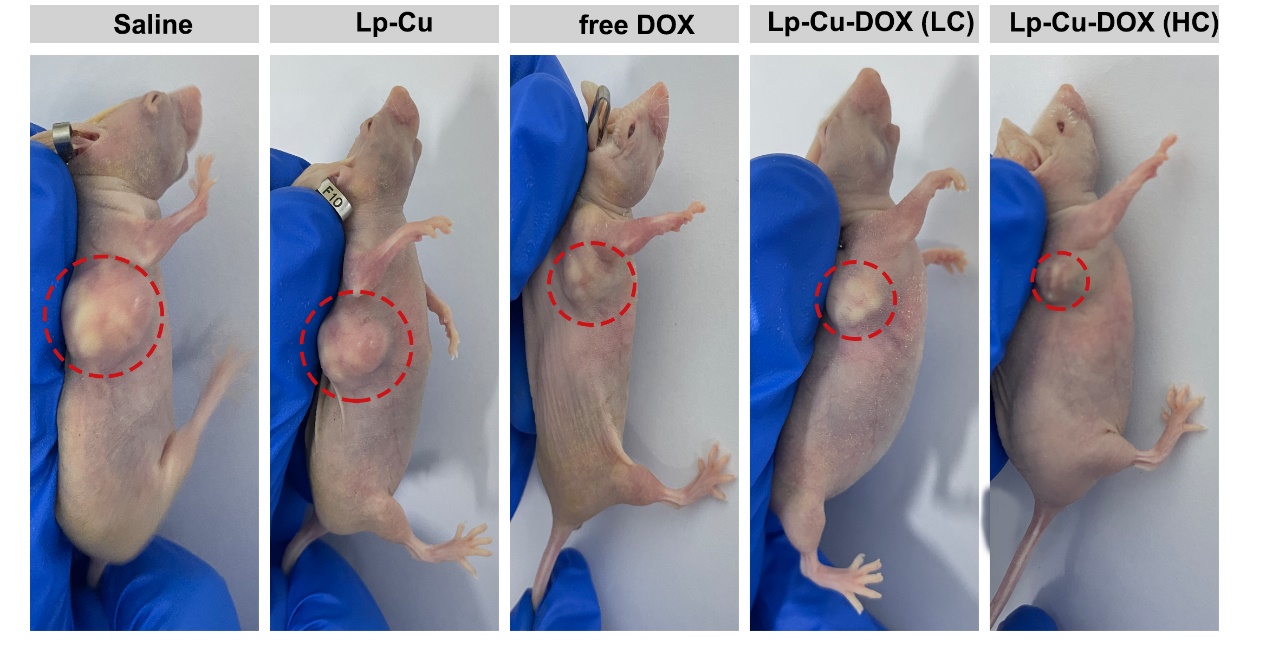


**Figure S5.** Photographs of tumor-bearing mice during the experiment.

**Figure S6.** Preliminary evaluation of in vivo safety: Body weight changes of different formulations.
